# Therapeutic benefits of gonadotropins in male hypogonadotropic hypogonadism: a focus on spermatogenesis and fertility

**DOI:** 10.1530/RAF-25-0100

**Published:** 2026-06-16

**Authors:** M Huijben, A M A Prinsen, C E de Keyser, S M van der Leij, E F H I Peeters, A M E Stades, S J Tanahatoe, L M O de Kort, H M K van Breda

**Affiliations:** ^1^ Department of Urology, University Medical Center Utrecht, Utrecht, The Netherlands; ^2^ Department of Endocrinology, University Medical Center Utrecht, Utrecht, The Netherlands; ^3^ Department of Gynaecology, University Medical Center Utrecht, Utrecht, The Netherlands

**Keywords:** azoospermia, gonadotropin therapy, hypogonadotropic hypogonadism, male infertility, male reproductive health

## Abstract

**Graphical Abstract:**

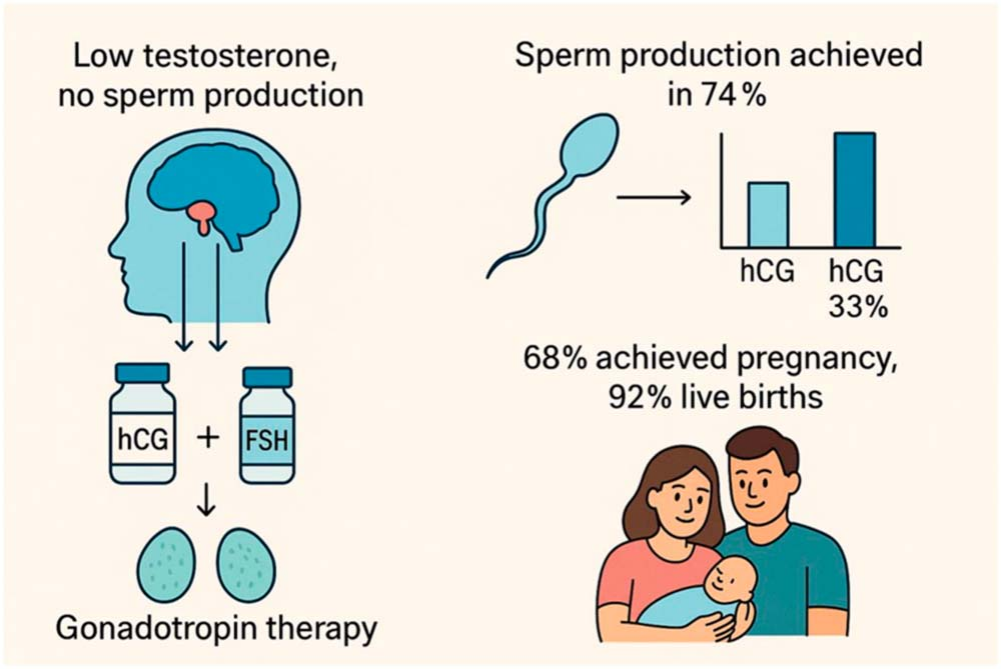

**Abstract:**

Male hypogonadotropic hypogonadism is characterized by deficient secretion of luteinizing hormone and follicle-stimulating hormone, leading to low testosterone levels, impaired spermatogenesis, and often infertility. Treatment aims to normalize testosterone and induce spermatogenesis, with gonadotropin therapy as standard. Factors affecting treatment success and optimal duration remain unclear. This single-center retrospective cohort study assessed the outcomes of gonadotropin therapy in men with hypogonadotropic hypogonadism and azoospermia. Men ≥ 18 years were included. The primary outcome was the successful induction of spermatogenesis. Data on demographics, treatment regimens, semen parameters, pregnancy outcomes, and side effects were collected. Regression analyses identified factors associated with treatment success. Thirty-five men were included. Spermatogenesis was achieved in 74% of patients, with higher success rates in those receiving combined human chorionic gonadotropin and follicle-stimulating hormone therapy (78%) versus human chorionic gonadotropin monotherapy (33%). Of those producing sperm, 69% upgraded to a higher WHO semen category, with 29% reaching normospermia. The median maximum sperm concentration achieved during treatment was 8.3 million/mL (IQR: 1.9–36.3). A larger baseline testicular volume was associated with a higher success rate of spermatogenesis and a shorter time to pregnancy. Pregnancy was achieved in 68% of patients, with 39% through spontaneous conception. The median time to first spermatogenesis was 7 months and to pregnancy was 21 months. Mild side effects occurred in 16% of patients. Gonadotropin therapy is effective and well tolerated in inducing spermatogenesis and achieving pregnancy in azoospermic men with HH. These findings, based on a contemporary Western cohort, provide updated evidence that supports personalized counseling and underscore the importance of long-term treatment adherence for successful fertility restoration.

**Lay summary:**

Some men are infertile because their brain does not produce the hormones needed to make sperm. This condition is called hypogonadotropic hypogonadism. These men often have low testosterone and no sperm in their semen. In this study, we looked at whether hormone treatment could help these men produce sperm and have children. We studied 35 men who were given hormone injections at a Dutch university hospital. After treatment, 74% of them started producing sperm. Those who received a combination of two hormones had better results than those who received only one. Almost 70% of men with sperm had better sperm quality, and over a quarter reached normal sperm levels. Two-thirds of men who wanted children became fathers, mostly through natural conception. Our findings show that hormone treatment is safe and often successful. This can offer hope to men with this condition who want to become fathers.

## Introduction

Hypogonadotropic hypogonadism (HH) is a condition characterized by the insufficient secretion of gonadotropins, specifically luteinizing hormone (LH) and follicle-stimulating hormone (FSH), from the anterior pituitary gland. In men, this leads to reduced testosterone production, resulting in symptoms such as reduced libido, fatigue, decreased testicular volume, diminished secondary sexual characteristics, erectile dysfunction, muscle weakness, and decreased body hair (Salonia *et al.* 2021). HH can also cause impaired spermatogenesis, leading to male infertility (Salonia *et al.* 2021). HH can be either congenital, often due to genetic mutations affecting the hypothalamic–pituitary–gonadal axis, as in Kallmann syndrome, or acquired, resulting from factors such as pituitary tumors, traumatic brain injury, systemic illnesses, hyperprolactinemia, anabolic steroid use, obesitas, and pituitary neoplasms (Salonia *et al.* 2021).

The main objective of HH treatment is to restore normal testosterone levels and stimulate spermatogenesis. Various fertility treatments are available for male HH patients who have a child wish, including clomiphene citrate, pulsatile gonadotropin-releasing hormone (GnRH) therapy, and gonadotropin-replacement therapy (Rastrelli *et al.* 2014). In patients with idiopathic hypogonadism, the first step in non-invasive treatment is clomiphene citrate, which is relatively low in cost (Huijben *et al.* 2023). If this proves ineffective, GnRH or gonadotropin therapy may be considered. However, these options are more expensive and require specialized devices for pulsatile administration (Dwyer *et al.* 2015). Both pulsatile GnRH and exogenous gonadotropin therapy yield similar outcomes (Liu *et al.* 1988, Schopohl *et al.* 1991). According to the European Association of Urology (EAU) Guideline on Sexual and Reproductive Health, gonadotropin therapy is recommended as the standard fertility treatment for men with HH (Salonia *et al.* 2021). LH is not suitable for clinical use due to its short half-life (Salonia *et al.* 2021). However, human chorionic gonadotropin (hCG) mimics LH and stimulates Leydig cells in the testes, promoting testosterone production. In addition, FSH directly stimulates Sertoli cells to support and maintain spermatogenesis (Dwyer *et al.* 2015, Prior *et al.* 2018, Salonia *et al.* 2021).

Several small retrospective observational studies and reviews have examined the effects of hCG/FSH therapy on infertile males with HH, and a recent systematic review (2024) on this subject focuses only on pubertal induction (Rastrelli *et al.* 2014, Young *et al.* 2019, Alexander *et al.* 2024). The overall success rate regarding improvement of spermatogenesis varies across studies, as does the reported pregnancy success rate (Rastrelli *et al.* 2014, Young *et al.* 2019). Individual responses to treatment can differ significantly, influenced by factors such as patient age, duration of HH, and baseline testicular function. Factors influencing the success rate or potential risk factors for therapy failure of hCG/FSH therapy are inconsistent and have not been clearly established (Rastrelli *et al.* 2014, Rohayem *et al.* 2016, Young *et al.* 2019). Moreover, few studies have provided detailed analyses of sperm quality progression or clinical predictors of fertility success, and the clinical meaning of improvements in sperm category for natural conception remains underexplored. Addressing these knowledge gaps is essential for guiding patient counseling and optimizing treatment strategies.

This retrospective study aims to evaluate the clinical outcomes of hCG and FSH therapy in men diagnosed with HH by assessing hormonal levels, spermatogenesis, and especially fertility.

## Methods

### Study design

This study was a retrospective cohort analysis conducted at a single center, aimed at evaluating the clinical outcomes of therapy with hCG and FSH therapy in men diagnosed with HH with infertility. The study was conducted at the University Medical Center (UMC) Utrecht from 2008 to 2022. Ethical approval for this study was obtained from the Local Research Ethics Committee, UMC Utrecht, the Netherlands (WAG/mb/22-908/DB).

### Study population

Men aged ≥ 18 years with HH and azoospermia, treated with hCG, with or without FSH, were included. Patients were excluded if no semen analysis was performed during follow-up or if hyperprolactinemia was identified as the cause of HH. Patients received hCG at doses ranging between 500 and 4,500 IU/week and recombinant or urinary FSH (Gonal-F and Bemfola) at doses ranging between 225 and 450 IU/week. Pregnyl® was initially used as hCG medication, but Ovitrelle® was utilized in the last years of study due to supply issues with Pregnyl®. FSH treatment was initiated once testosterone levels were normalized with hCG. Testicular volume was measured with an orchidometer, ultrasound, or both.

### Outcomes

Medical records of eligible patients were reviewed to extract relevant data. The primary outcome was the success rate of gonadotropin-induced spermatogenesis, defined as the percentage of male HH patients treated with hCG who exhibited the presence of at least one spermatozoon in the ejaculate.

Secondary outcomes were hormonal levels, sperm parameters and concentrations, pregnancy rates, and self-reported side effects during gonadotropin treatment. Sperm concentrations were classified according to WHO fertility guidelines as follows: azoospermia (0 million/mL), severe oligozoospermia (0–5 million/mL), moderate oligozoospermia (5–10 million/mL), mild oligozoospermia (10–15 million/mL), and normozoospermia (>15 million/mL) (Cooper *et al.* 2009).

### Statistical analysis

Data were analyzed using SPSS (IBM SPSS Statistics, USA, version 25.0). Descriptive statistics are presented as counts (%), means (SD), or medians (IQR). Statistical significance was set at *P* ≤ 0.05. The success rate of hCG-induced spermatogenesis was evaluated by the presence of spermatozoa, defined as the proportion of treated men with at least one spermatozoon present at semen analysis. Paired *t*-tests or Wilcoxon (non-normal) signed rank tests were used for paired continuous data. Independent *t*-tests (normal data) and Mann–Whitney U tests (non-normal data) were used for continuous non-paired data. Chi-squared tests were performed for categorical data (Fisher’s exact test in case of low frequencies (*n* < 5)). Univariate and multivariate Cox regression analyses were performed to assess the association between patient- and treatment-related factors and the time to spermatogenesis. Backward stepwise selection was used in multivariate models, with variables showing a *P*-value < 0.1 in univariate analysis considered for inclusion. A *P*-value < 0.05 was considered statistically significant in the final multivariate model. Multicollinearity was assessed using variance inflation factor (VIF); variables with VIF > 5 were closely examined, and those showing high redundancy or conceptual overlap were excluded from the final model. Kaplan–Meier survival analysis and log-rank tests were used to assess the time to spermatogenesis. For this analysis, patients were grouped based on testicular volume into ‘small’ and ‘large’ volume categories using 4 mL as a cutoff value, in accordance with the existing literature (Dwyer *et al.* 2015, Prior *et al.* 2018).

## Results

### Study population

Of the 69 patients treated with gonadotropins, 34 were excluded for being hypergonadotropic (*n* = 4), not azoospermic (*n* = 11), or lacking a semen analysis (*n* = 19). In total, 35 patients were included for analysis, and divided into subgroups of spermatogenesis and no spermatogenesis during treatment (Table 1). The mean age of patients at the start of therapy was 31.5 ± 4.2 years, which is comparable to, though slightly lower than, the mean paternal age in the Netherlands in 2024 (34.5 years) (CBS 2025, available at: https://www.cbs.nl/nl-nl/nieuws/2025/24/77-duizend-mannen-voor-het-eerst-vader-in-2024). The mean duration of the hCG therapy was 34.4 ± 3.4 months, and that of the FSH therapy was 25.8 ± 3.2 months. FSH addition started in 18 patients after hCG, in 14 patients simultaneously with hCG treatment, and in 3 patients the timing of FSH addition was unknown. No patients had varicocele, orchidectomy, or radiotherapy in their medical history. One patient had a history of chemotherapy. Eighty percent had previously received testosterone therapy (TTh) for a mean of 93.3 ± 11.2 months. Of the 13 patients reporting changes in testicular volume during gonadotropin therapy, 62% (8/13) noticed an increase during treatment, the other patients did not report this outcome or did not notice a change in volume. Two men had ultrasound measurements showing a doubling of their initial testicular volume during the therapy. Thirty-two patients had a female partner during the study, and 28 expressed a desire to have a child. The mean age of the female partners was 27.4 ± 9.3 years. None of the female partners reported fertility issues.

**Table 1:** Patient characteristics at baseline of the total population and comparison between patients with spermatogenesis and without spermatogenesis during gonadotropin therapy. Data are presented as *n* (%) of patients, mean ± SD or as median (IQR).

	Total (*n* = 35)	Spermatogenesis (*n* = 26)	No spermatogenesis (*n* = 9)	*P*-value
Age at the start of therapy	31.5 ± 4.2	31.1 ± 4.5	32.7 ± 4.0	0.335
BMI (kg/m^2^)	24 (23–30)	24.5 (22–31.2)	24 (23–25.5)	0.725
Cause of HH				<0.001*
Kallman	5 (14)	0	5 (56)	
Testosterone	4 (11)	4 (15)	0	
Panhypopit after resection macroadenoma	7 (20)	6 (23)	1 (11)	
Idiopathic	10 (29)	9 (35)	1 (11)	
Congenital panhypopit	5 (14)	5 (19)	0	
Secondary hypogonadism	1 (3)	1 (4)	0	
Dax mutation	2 (6)	0	2 (22)	
Head trauma	1 (3)	1 (4)	0	
Onset of HH				0.135
Pre-pubertal	19 (54)	12 (46)	7 (78)	
Post-pubertal	16 (46)	14 (54)	2 (22)	
Previous treatment				
Gonadotropins	10 (29)	6 (23)	4 (44)	0.393
TTh	28 (80)	20 (77)	8 (89)	0.648
Corticosteroids	9 (26)	8 (31)	1 (11)	0.391
Cryptorchidism in the past	4 (11)	1 (4)	3 (33)	0.044*
Baseline testicular volume (mL)	4 (2.6–10)	8.2 (3–10.2)	2.7 (2–6.8)	0.049*
Treatment duration (months)				
hCG	34 ± 20	36 ± 20	28 ± 19	0.304
FSH	26 ± 17	29 ± 17	12 ± 6	<0.001*

BMI, body mass index; TTh, testosterone therapy; HH, hypogonadotropic hypogonadism.

* *P* ≤ 0.05.

### Spermatogenesis

**Figure 1:**
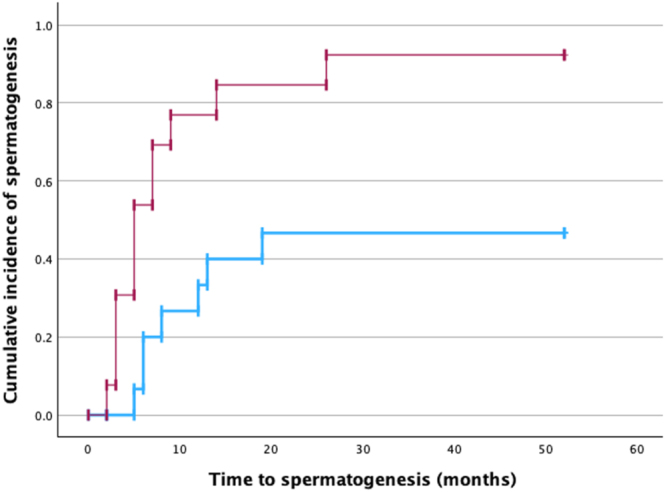
Kaplan–Meier curve – cumulative probability of spermatogenesis stratified by baseline testicular volume over time (months). The blue line indicates patients with a smaller testicular volume at baseline <4 mL, and the red line indicates patients with a larger testicular volume at baseline of ≥4 mL.

Spermatogenesis was achieved by 74% of the patients (26/35), 78% in the hCG + FSH therapy group (25/32), and 33% in the hCG monotherapy group (1/3). Further details on spermatogenesis are presented in Table 1. Spermatogenesis was achieved in 63% (12/19) of patients with pre-pubertal onset HH, compared to 87% (14/16) of those with post-pubertal onset HH (Table 1). When stratified by baseline testicular volume, patients with larger testes (≥4 mL) had a spermatogenesis rate of 92%, compared to 47% in those with smaller testes (<4 mL) (Fig. 1, *P* = 0.041). The median baseline testicular volume was three times higher in patients who achieved spermatogenesis compared to those who did not (*P* = 0.049). Cryptorchidism was eight times more prevalent among patients who did not achieve spermatogenesis (*P* = 0.044). In univariate analysis (Table 2), larger baseline testicular volume and longer duration of FSH therapy were positively associated with achieving spermatogenesis (*P* = 0.086 and *P* = 0.059, respectively), while the presence of cryptorchidism was negatively associated (*P* = 0.042). Cryptorchidism was excluded from the multivariate analysis due to quasi-complete separation, possibly resulting from both its strong association with the outcome and its low prevalence in the dataset, a statistical issue that prevented the model from generating stable or interpretable estimates. In the multivariate model, testicular volume (*P* = 0.062) and FSH therapy duration (*P* = 0.083) remained potential predictors, though not statistically significant.

**Table 2:** Univariate and multivariate logistic regression on achieving spermatogenesis.

Variable	Univariate			Multivariate		
	B	OR (95%CI)	*P*-value	B	OR (95%CI)	*P*-value
Age at the start of therapy (years)	−0.09	0.92 (0.76–1.10)	0.346	-	-	-
BMI (kg/m^2^)	0.08	1.09 (0.90–1.31)	0.386	-	-	-
Baseline FSH (IU/L)	−2.48	0.08 (0.00–3.42)	0.190	-	-	-
Testicular volume (mL)	0.21	1.24 (0.97–1.58)	0.086	0.36	1.44 (0.98–2.11)	0.062
Onset of HH				-	-	-
Pre-pubertal (ref.)						
Post-pubertal	1.41	4.08 (0.71–23.51)	0.115			
Cryptorchidism				-	-	-
Yes (ref.)						
No	−2.53	0.08 (0.01–0.91)	0.042*			
Duration of therapy (months)						
Gonadotropin	0.03	1.03 (0.98–1.07)	0.292	-	-	-
FSH	0.012	1.13 (0.99–1.28)	0.059	0.18	1.19 (0.98–1.46)	0.083

BMI, body mass index; HH, hypogonadotorpic hypogonadism; FSH, follicle-stimulating hormone; OR, odds ratio; CI, confidence interval; ref., reference group.

* *P* ≤ 0.1.

### Time to spermatogenesis

**Figure 2:**
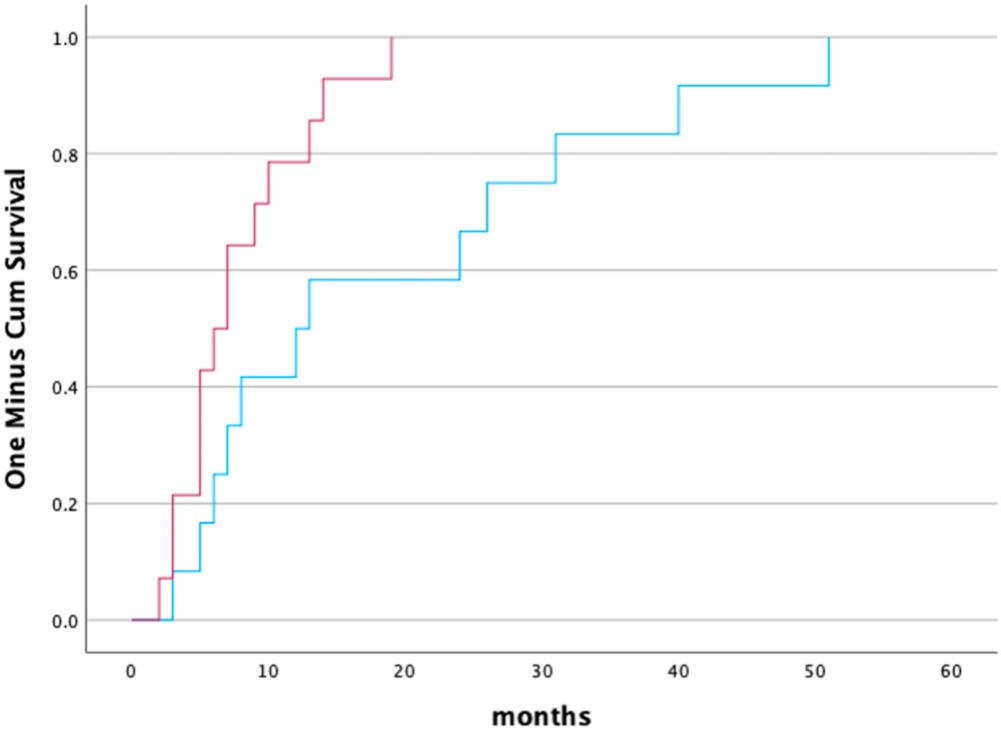
Kaplan–Meier curve – cumulative probability of spermatogenesis stratified by onset moment of hypogonadotropic hypogonadism over time (months). The blue line indicates patients with pre-pubertal onset of HH, and the red line indicates post-pubertal onset of HH.

The median time to first spermatogenesis was 7 months (5–13), while the mean time to reach maximum sperm concentration was 24.9 ± 3.7 months. The duration of FSH treatment was 2.5 times longer in patients who achieved spermatogenesis (*P* < 0.001). The time to first sperm differed between pre- and post-pubertal onset of HH (*P* = 0.035), with a median of 8 months (6–26) for pre-pubertal onset of HH and a median of 6 months (4–10) for post-pubertal onset of HH (Fig. 2). The time to first spermatogenesis did not differ between patients receiving FSH simultaneously with hCG and those receiving FSH after initiation of hCG therapy, neither in the pre-pubertal (*P* = 0.074) nor in the post-pubertal onset group (*P* = 0.531). In univariate Cox regression analysis on time to spermatogenesis (Table 3), a larger testicular volume was significantly associated with a higher likelihood of achieving earlier spermatogenesis (*P* = 0.002). Post-pubertal onset of HH (vs pre-pubertal) was also associated with a significantly higher hazard (*P* = 0.004). The presence of cryptorchidism showed a trend toward a longer time to achieve spermatogenesis (*P* = 0.073). In multivariate analysis, only testicular volume remained significantly associated with time to spermatogenesis (*P* = 0.007). Cryptorchidism was excluded from multivariate analysis due to estimation instability, likely resulting from sparse data or a lack of events in one category, which prevents reliable hazard estimation.

**Table 3:** Cox regression analysis on time to first spermatogenesis (months).

Variable	Univariate			Multivariate		
	HR	95% CI	*P*-value	HR	95% CI	*P*-value
Age at the start of therapy (years)	0.99	0.90–1.09	0.821	-	-	-
BMI (kg/m^2^)	1.03	0.94–1.13	0.497	-	-	-
Baseline FSH (IU/L)	0.25	0.04–1.72	0.159	-	-	-
Testicular volume (mL)	1.30	1.10–1.53	0.002*	1.27	1.07–1.51	0.007^†^
Onset of HH						
Pre-pubertal (ref.)						
Post-pubertal	3.84	1.53–9.66	0.004*	0.82	0.27–2.53	0.728
Cryptorchidism				-	-	-
Yes	0.16	0.02–1.19	0.073*			
No (ref.)						

HH, hypogonadotropic hypgonadism; BMI, body mass index; FSH, follicle-stimulating hormone; ref., reference group; HR, hazard ratio.

* *P* < 0.1.

^†^ *P* < 0.05.

### Other sperm parameters

For all sperm parameters of the entire group, as well as for the subgroups divided by pre- and post-pubertal onset of HH, see Table 4. Among the 26 patients who achieved spermatogenesis, sperm concentrations upgraded to severe oligozoospermia in 29% (*n* = 10), moderate oligozoospermia in 9% (*n* = 3), mild oligozoospermia in 3% (*n* = 1), and normozoospermia in 29% (*n* = 10). Univariate linear regression (Table 5) identified baseline testicular volume as the only significant predictor of total motile sperm count (*P* = 0.034), while the duration of gonadotropin and FSH therapy showed borderline significance (*P* = 0.059 and *P* = 0.075, respectively). No predictors remained significant in multivariate analysis.

**Table 4:** Sperm parameters at first and maximum sperm concentration and spontaneous pregnancy during gonadotropin treatment. Data are presented as mean ± SD or as median (IQR).

	Sperm parameters at first appearance			Sperm parameters at maximum concentration			Sperm parameters at SP
	Total (*n* = 35)	Pre-pubertal (*n* = 19)	Post-pubertal (*n* = 16)	Total (*n* = 25)	Pre-pubertal (*n* = 12)	Post-pubertal (*n* = 13)	Total (*n* = 8)
Volume (mL)	2.6 ± 1.5	2.5 ± 1.7	2.7 ± 1.2	3.2 ± 1.5	3.1 ± 1.9	3.3 ± 1.1	3.5 ± 1.2
Sperm concentration (million/mL)	0.1 (0.0–5.4)	0.0 (0.0–4.4)	0.5 (0.03–8)	8.3 (1.9–36.3)	8.3 (1.3–30.8)	11.2 (1.8–39)	3.1 (1.2–34.5)
Progressive motility (%)	34 ± 23	34 ± 25	34 ± 23	43 ± 21	31 ± 21	53 ± 16*	41 ± 23
Total motility (%)	34 ± 26	35 ± 28	34 ± 26	48 ± 21	41 ± 19	55 ± 21	39 ± 25
Morphology (%)	4 ± 3	5 ± 4	3 ± 3	2 (1–3.5)	2 (0.0–4.5)	3 (2–3)	1 (1–2)
Total motile sperm count	0.1 (0.0–3.1)	0.0 (0.0–0.6)	0.3 (0.1–4.4)	14.6 (3.0–54.7)	10.7 (1.1–47.8)	19.7 (3.1–68.6)	9.7 (1–79.5)

SP, spontaneous pregnancy.

* *P* = 0.011 (≤0.05).

**Table 5:** Univariate and multivariate linear regression on total motile sperm count.

Variable	Univariate			Multivariate		
	B (95%CI)	SE	*P*-value	B (95%CI)	SE	*P*-value
Age at the start of therapy (years)	2.34 (−3.75 to 8.43)	2.95	0.436	-	-	-
BMI (kg/m^2^)	−1.22 (−6.89 to 4.44)	2.74	0.660	-	-	-
Baseline FSH (IU/L)	85.66 (−47.76 to 219.09)	64.34	0.197	-	-	-
Testicular volume (mL)	8.62 (0.74 to 16.50)	3.72	0.034*	6.47 (−6.78 to 19.73)	6.08	0.308
Onset of HH				-	-	-
Pre-pubertal (ref.)						
Post-pubertal	22.38 (−31.26 to 76.01)	25.99	0.398			
Cryptorchidism				-	-	-
Yes (ref.)						
No	112.92 (−19.99 to 245.84)	64.40	0.092			
Duration of therapy (months)						
Gonadotropin	−1.28 (−2.61 to 0.52)	0.64	0.059*	0.99 (−11.57 to 13.54)	5.76	0.867
FSH	−1.50 (−3.17 to 0.16)	0.80	0.075*	−2.85 (−15.50 to 9.81)	5.81	0.633

HH, hypogonadotropic hypgonadism; BMI, body mass index; FSH, follicle-stimulating hormone; ref., reference group.

* *P* ≤ 0.1.

### Achieving pregnancy

Among the 28 patients with a pregnancy wish, 68% (19/28) achieved a total of 25 pregnancies during the study period. Conception occurred spontaneously in 39% (11/28), through intrauterine insemination (IUI) in 7% (2/28), and via intracytoplasmic sperm injection (ICSI) in 21% (6/28). Seven patients did not achieve pregnancy, one was lost to follow-up, and one had just initiated gonadotropin therapy, with successful sperm cryopreservation after the first semen analysis. Of the 25 pregnancies, 92% (*n* = 23) resulted in live births, one ended in miscarriage, and the outcome of one pregnancy was unknown. Sperm parameters associated with spontaneous conception are presented in Table 4. The cryopreservation success rate was 86% (18/21 couples). In univariate logistic regression (Table 6), a longer duration of FSH therapy when TT levels were normalized was significantly associated with higher odds of achieving pregnancy (*P* = 0.034). Baseline FSH levels, duration of gonadotropin therapy, and age at treatment initiation showed borderline associations (*P* = 0.073, *P* = 0.053, and *P* = 0.099, respectively). In multivariate analysis, none of these variables remained statistically significant.

**Table 6:** Univariate and multivariate logistic regression on achieving pregnancy.

Variable	Univariate			Multivariate		
	B	OR (95%CI)	*P*-value	B	OR (95%CI)	*P*-value
Age at the start of therapy (years)	−0.21	0.80 (0.62–1.04)	0.099*	−115.96	0.0 (0.0)	0.976
BMI (kg/m^2^)	0.25	1.28 (0.92–1.77)	0.137	-	-	-
Baseline FSH (IU/L)	−4.25	0.01 (0.00–1.49)	0.073*	−1,550.85	0.0 (0.0)	0.975
Testicular volume (mL)	0.05	1.05 (0.84–1.30)	0.670	-	-	-
Onset of HH				-	-	-
Pre-pubertal	0.39	1.48 (0.26–8.50)	0.659			
Post-pubertal (ref.)						
Duration of therapy (months)						
Gonadotropin	0.07	1.08 (0.99–1.16)	0.053*	0.13	1.14 (0.00–1.72)	0.999
FSH	0.13	1.14 (1.01–1.28)	0.034*	−2.77	0.06 (0.0–1.44)	0.987

HH, hypogonadotropic hypgonadism; BMI, body mass index; FSH, follicle-stimulating hormone; ref., reference group.

* *P* ≤ 0.1 in univariate analysis.

### Time to pregnancy

The median time to pregnancy from therapy initiation was 21 (IQR: 18–30) months, and the mean time from first evidence of spermatogenesis to pregnancy was 12.2 ± 2.2 months. In univariate Cox regression (Table 7), older age at treatment initiation, larger baseline testicular volume, and post-pubertal onset of HH were significantly associated with shorter time to pregnancy (*P* = 0.004, *P* = 0.033, and *P* = 0.008). In the multivariate Cox regression analysis, both older age at the start of therapy (*P* = 0.007) and greater baseline testicular volume (*P* = 0.006) remained independently associated with a higher probability of achieving pregnancy over time. The variable onset of HH was excluded from the multivariate model due to estimation instability and complete separation observed in earlier models. This likely reflects sparse data or an absence of pregnancy events in one of the onset categories, which prevented reliable hazard estimation.

**Table 7:** Cox regression on time to pregnancy in months from the start of therapy.

Variable	Univariate			Multivariate		
	HR	95% CI	*P*-value	HR	95% CI	*P*-value
Age at the start of therapy (years)	1.108	1.03–1.19	0.004*	1.29	1.07–1.56	0.007^†^
BMI (kg/m^2^)	1.08	0.98–1.20	0.128	-	-	-
Baseline FSH (IU/L)	0.62	0.08–4.57	0.637	-	-	-
Testicular volume (mL)	1.15	1.01–1.31	0.033*	1.44	1.11–1.88	0.006^†^
Onset of HH				-	-	-
Pre-pubertal (ref.)						
Post-pubertal	5.94	1.58–22.34	0.008*			

HH, hypogonadotropic hypgonadism; HR, hazard ratio; BMI, body mass index; FSH, follicle-stimulating hormone; ref., reference group.

* *P* ≤ 0.1.

^†^ *P* ≤ 0.05.

### Hormonal levels

Table 8 presents the hormonal values for all patients, stratified by pre- and post-pubertal onset of HH. The median time to achieve adequate serum testosterone levels during gonadotropin therapy was 6 weeks (4–12). Among the 28 patients in whom testosterone was measured during treatment, 89% (25/28) reached levels above the diagnostic threshold for hypogonadism (>12.1 nmol/L). Mean LH remained suppressed (<1 IU/L), and mean FSH was 3.6 IU/L. No significant differences in hormonal levels were observed between the pre- and post-pubertal onset groups.

**Table 8:** Hormonal evaluation at baseline, at first, and at maximum sperm concentration during gonadotropin treatment. Data are presented as mean ± SD or median (IQR).

	All patients (*n* = 35)		Pre-pubertal onset (*n* = 19)		Post-pubertal onset (*n* = 16)	
	*n*	Values	*n*	Values	*n*	Values
Total testosterone (nmol/L)						
Baseline	32	3.5 (0.7–10.8)	17	3.5 (0.7–8.1)	15	3.6 (0.7–12.0)
First spermatogenesis	29	23.9 ± 1.8	14	25.1 ± 2.4	15	22.8 ± 1.8
Maximum sperm concentration	29	25.2 ± 2.3	12	28.4 ± 3.4	13	22.3 ± 3.1
FSH (IU/L)						
Baseline	29	0.6 ± 0.4	15	0.5 (0.5–0.5)	14	0.5 (0.5–0.8)
First spermatogenesis	22	3.4 ± 0.5	8	3.2 ± 0.9	14	3.3 (2.3–4.1)
Maximum sperm concentration	13	3.6 (3.0–5.2)	4	4.7 (3.8–5.9)	9	3.3 (2.8–4.4)
LH (IU/L)						
Baseline	29	0.5 (0.5–0.5)	15	0.5 (0.5–0.6)	14	0.5 (0.5–0.5)
First spermatogenesis	20	0.5 (0.5–0.5)	7	0.5 (0.3–0.5)	13	0.5 (0.5–0.5)
Maximum sperm concentration	13	0.5 (0.4–0.5)	4	0.5 (0.3–0.5)	9	0.5 (0.4–0.5)

FSH, follicle-stimulating hormone; LH, luteinizing hormone; *n*, number of patients.

### Side effects

In the patients who reported side effects, 16% (3/18) experienced mild symptoms from gonadotropin treatment. Two patients reported gynecomastia while using Pregnyl, and one patient complained about hot flushes after switching from Pregnyl to Ovitrelle. The patients found the side effects mild and continued treatment. There was no correlation between hormonal levels during therapy and the presence of side effects.

## Discussion

This study showed that gonadotropin therapy, especially combining hCG and FSH, effectively induces spermatogenesis in patients with HH. Spermatogenesis was achieved in 74% of patients, with higher success rates in combined therapy compared to hCG monotherapy. In addition, 69% of patients had an upgrade in sperm category (according to the WHO guidelines). Larger baseline testicular volume was associated with shorter time to spermatogenesis, higher spermatogenesis rate, and shorter time to pregnancy. Our findings expand the limited recent literature by providing detailed insight into both quantitative and qualitative improvements in spermatogenesis, including upgrades in sperm category, and by linking these outcomes to clinically meaningful fertility results such as pregnancy achievement. Importantly, our cohort reflects the current clinical population, in which men tend to seek fertility treatment at a later age, underscoring the real-world applicability of these results.

Spermatogenesis was achieved in 74% of patients, a notable outcome compared to previous studies with success rates ranging from 48 to 75% (Burgués & Calderón 1997, Abbasi *et al.* 2008, Rohayem *et al.* 2016, Chen *et al.* 2020, Yılmazel *et al.* 2021). The success rate was higher among patients treated with the combined hCG and FSH therapy (78%) compared to those receiving only hCG monotherapy (33%); this aligns with previous studies demonstrating spermatogenesis rates of 25–33% with monotherapy and 50–100% with combination therapy (Fuse *et al.* 1996, Zacharin *et al.* 2012). Baseline testicular volume was approximately three times higher in patients who achieved spermatogenesis. Although univariate analysis demonstrated a positive association between larger testicular volume and achieving spermatogenesis, this association did not remain in multivariate analysis. Stratification by testicular volume showed that 92% of patients with a baseline volume ≥ 4 mL achieved spermatogenesis, compared to 47% in those with smaller testes (<4 mL). Although subgroup sizes were too small for inclusion in regression models, this observation supports prior studies indicating that greater testicular volume correlates with improved sperm concentration and likelihood of spermatogenesis (Schopohl *et al.* 1991, VICARI *et al.* 1992, Kung *et al.* 1994, Burgués & Calderón 1997). Similarly, the sample size for patients with and without cryptorchidism was insufficient for multivariate analysis. However, in univariate analysis, the absence of cryptorchidism was positively associated with spermatogenesis, consistent with previous findings (Bouvattier *et al.* 1999, Liu *et al.* 2019). Interestingly, none of the patients with Kallmann syndrome (*n* = 5) achieved spermatogenesis in our cohort. This contrasts with prior studies reporting success rates between 70 and 100% in this subgroup when treated with gonadotropins (Depenbusch *et al.* 2002; ‘Efficacy and safety of highly purified urinary follicle-stimulating hormone with human chorionic gonadotropin for treating men with isolated HH’, 1998). A possible explanation could be the duration of gonadotropin therapy as in a previous study with 111 Kallmann patients, a longer treatment duration of at least 18 months was suggested, whereas in our study, the median duration of therapy among Kallmann patients was only 12 months (Liu *et al.* 2016). Taken together, these findings emphasize the importance of early identification of favorable prognostic factors, such as larger testicular volume and absence of cryptorchidism, and suggest that extended treatment duration may be particularly critical for patients with Kallmann syndrome.

The median time to first spermatogenesis in our study was 7 months, which is in line with previous studies showing median times of 5, 8.5, and 9 months (Burgués & Calderón 1997, Zorn *et al.* 2005, Zhang *et al.* 2015). Liu *et al.* (2016) reported that the time to first spermatogenesis could extend up to 42 months, underscoring the importance of long-term follow-up and patient counseling (Liu *et al.* 2016). Such information is crucial in clinical practice to set realistic expectations regarding the timeframe for achieving spermatogenesis. In our cohort, the time to first spermatogenesis was shorter in patients with post-pubertal onset of HH (median: 6 months) compared to those with pre-pubertal onset of HH (median 8 months). This difference is consistent with findings from Zorn *et al.* (2005), who demonstrated a median time of 2.5 months in post-pubertal onset of HH versus 14.5 months in pre-pubertal onset of HH (Zorn *et al.* 2005). The more favorable outcomes in patients with post-pubertal onset may be explained by underlying physiological differences. As suggested by Bouvattier *et al.* (2012), patients with acquired (post-pubertal) HH often have a larger baseline testicular volume and a more preserved reservoir of Leydig and Sertoli cells, both of which are essential for successful spermatogenesis (Bouvattier *et al.* 2012). In our univariate Cox regression analysis, smaller baseline testicular volume, pre-pubertal onset of HH, and cryptorchidism were associated with a delayed time to spermatogenesis. However, in multivariate analysis, only a larger baseline testicular volume remained significantly associated with a higher likelihood of earlier spermatogenesis. This finding aligns with the study of Ma *et al.* (2021), showing that a larger testicular size at baseline was a favorable indicator for earlier spermatogenesis (Ma *et al.* 2021). These findings reinforce the clinical value of baseline testicular volume as a prognostic marker for timing of spermatogenesis in HH patients. Importantly, our findings also suggest that initiating FSH therapy simultaneously with LH may not be necessary. In our cohort, delaying the start of FSH until serum testosterone levels had normalized did not appear to adversely affect the time to first spermatogenesis. This observation has potential clinical implications, as postponing FSH initiation could substantially reduce treatment costs without compromising treatment outcomes.

The median maximum sperm concentration achieved during treatment was 8.3 million/mL (1.9–36.3), which is comparable to previous studies reporting concentrations between 8 and 24.5 million/mL in previously azoospermic men undergoing gonadotropin (Burgués & Calderón 1997, Abbasi *et al.* 2008, Rohayem *et al.* 2016). Of the 26 patients who achieved spermatogenesis during therapy, 69% upgraded to a higher WHO sperm category, with 29% reaching normospermia. During treatment, the median sperm concentration was 3.1 million/mL (1.2–34.5) and the median total motile sperm count at the time of spontaneous conception was 9.7 million (1–79.5). According to the WHO 2010 guidelines used during the study period, a TMSC > 15 million/mL is considered within the normal range for natural conception (Cooper *et al.* 2009). This highlights that even subthreshold sperm parameters can be sufficient for natural conception in this population, which may help guide more individualized fertility counseling.

In this study, 68% of patients with a child wish achieved at least one pregnancy during gonadotropin therapy, with a high live birth rate of 92%. This is comparable with previous studies, where pregnancy rates ranged between 20 and 70% (Burgués & Calderón 1997, Chen *et al.* 2021; ‘Efficacy and safety of highly purified urinary follicle-stimulating hormone with human chorionic gonadotropin for treating men with isolated HH’, 1998). Initial pregnancies were achieved through spontaneous conception in 39% out of patients with a child wish, which is comparable to studies with larger sample sizes (*n* > 35), reporting rates of 33, 70, 37, and 52% (Abbasi *et al.* 2008, Rohayem *et al.* 2016, Ortac *et al.* 2019, Yılmazel *et al.* 2021). However, in these studies, it was often unclear whether the outcomes referred to the proportion of patients with a child wish or to total pregnancies (including assisted reproductive technologies). In summary, our findings reinforce that gonadotropin therapy can lead to high pregnancy and live birth rates in men with HH, but achieving pregnancy generally requires sustained treatment, and patient counseling should reflect these timelines.

The median time to pregnancy from therapy initiation was approximately 21 months, aligning with previous findings (ranging between 10 and 37 months), suggesting that spermatogenesis and subsequent conception often require prolonged treatment duration (Matsumoto *et al.* 2009, Rohayem *et al.* 2016, Ortac *et al.* 2019). Univariate Cox regression analyses indicated that older age at therapy initiation, larger baseline testicular volume, and post-pubertal onset of HH were significantly associated with a shorter time to pregnancy. In the multivariate model, both older age and larger baseline testicular volume remained significant independent predictors. These findings suggest that men who begin therapy at an older age and those with greater initial testicular volume may have a more favorable prognosis regarding fertility outcomes. These findings align with prior literature, who reported that larger testicular volume and older age were associated with improved fertility outcomes (Liu *et al.* 2016, Ortac *et al.* 2019). The reason for older age being a predictor is unknown; however, Liu *et al.* (2016) suggest that this could perhaps be due to being more motivated to engage in therapy in patients at older age. Our results underscore the potential importance of patient age and testicular reserve in post-pubertal men, predicting reproductive success among men with HH.

Side effects (gynecomastia and hot flushes) were reported in 16% of patients. The literature reports prevalence of side effects ranging between 6 and 52%, mostly hot flushes, gynecomastia, breast tenderness, and acne (Schopohl *et al.* 1991, Anawalt *et al.* 1996, Matsumoto *et al.* 2009). A study of Zhang *et al.* (2019) involving 28 patients suggested a tendency between high serum testosterone levels and the presence of acne and breast tenderness. However, in our cohort, we did not observe such an association.

This study has several limitations, including a relatively small sample size that reduced statistical power, especially for subgroup analyses. The retrospective design introduced potential selection and recall biases, and inconsistent documentation may have led to underreporting of side effects. Only a small number of patients in our cohort had a history of cryptorchidism (*n* = 4). Although cryptorchidism is a known adverse prognostic factor for spermatogenic recovery in congenital HH, the small subgroup size precluded reliable statistical evaluation and it was, therefore, not included in the multivariate analysis. Consequently, the potential impact of cryptorchidism on treatment response could not be adequately assessed. Variations in follow-up duration and timing of measurements may have influenced outcomes. In addition, the number and timing of semen analyses were not standardized due to the retrospective design, and complete data on the exact number of samples per patient were not consistently available. This variability in sampling frequency and intervals may have influenced the estimates of time to spermatogenesis and sperm concentration, potentially leading to variability in the observed response rates. Furthermore, our findings should be interpreted in the context of the existing literature. This study is partly cumulative and confirmatory, as gonadotropin therapy for male HH has been described previously. However, given the limited number of recent Western cohorts and the scarcity of studies reporting detailed fertility outcomes, our data contribute updated clinical evidence on spermatogenesis progression and pregnancy outcomes in contemporary practice. Finally, we did not assess improvement in hypogonadal symptoms, which represents an important aspect of treatment beyond fertility. Although testis volume was associated with spermatogenesis in univariate analysis, this did not persist in multivariate analysis, possibly reflecting residual confounding or insufficient statistical power.

Despite these limitations, this study provides valuable insights into spermatogenesis and pregnancy outcomes with gonadotropin therapy in HH patients, with a particular focus on identifying potential predictors for treatment success, and offers a novel comparison between pre- and post-pubertal onset of hypogonadism. It confirms the effectiveness of combination hCG and FSH therapy in inducing spermatogenesis and achieving pregnancies. Importantly, this study refines existing evidence by identifying baseline testicular volume and age at therapy initiation as predictors of spermatogenesis and time to pregnancy, underscoring the value of early, individualized assessment. The absence of spermatogenesis in patients with Kallmann syndrome, despite high success rates in previous studies, suggests that longer treatment durations may be needed in this subgroup. Notably, our findings also indicate that natural conception may occur at sperm concentrations below the WHO thresholds, challenging current assumptions about minimum semen parameters for fertility in HH. In addition, our results suggest that initiating FSH therapy simultaneously with LH may not always be necessary, as delaying FSH until testosterone levels have normalized did not appear to adversely affect the time to spermatogenesis. This approach may have important practical implications, as postponing FSH initiation could substantially reduce treatment costs without compromising treatment outcomes. In sum, this study confirms the effectiveness of gonadotropin therapy in restoring fertility, while highlighting testicular volume, age, and therapy duration as key prognostic factors to guide clinical decision-making. These findings are particularly relevant given the high cost and intensity of treatment, which necessitates careful consideration of the balance between financial burden and the likelihood of achieving a successful pregnancy. While not addressed in this study, lifestyle modifications, such as smoking cessation, maintaining a healthy BMI, limiting alcohol consumption, avoiding drugs/anabolics, and reducing scrotal heat exposure, are key elements in all subfertility guidelines. Their potential impact on treatment outcomes warrants attention, especially in light of limited healthcare resources and the importance of patient responsibility when choosing costly therapies.

## Conclusion

Our results confirm the high therapeutic efficacy and safety of gonadotropin therapy in achieving spermatogenesis and successful pregnancies in a contemporary cohort of azoospermic men with HH. Spermatogenesis was achieved in 74% of patients, and 68% of those with a child wish ultimately achieved pregnancy. Importantly, achieving pregnancy often requires prolonged treatment and dual therapy with FSH, underscoring the need for patience and continued therapy. A larger testicular volume was associated with a higher rate of achievement of spermatogenesis and an earlier time to spermatogenesis. Furthermore, a larger baseline testicular volume and older age at therapy initiation were predictors for a shorter time to pregnancy. These findings emphasize the value of these clinical predictors as tools for personalized counseling and setting realistic expectations about fertility restoration timelines and outcomes in men with HH.

## Declaration of interest

The authors declare that there is no conflict of interest that could be perceived as prejudicing the impartiality of the research reported.

## Funding

This research did not receive any specific grant from any funding agency in the public, commercial, or not-for-profit sector.

## Author contribution statement

All authors contributed substantially to the conception, design, data acquisition, analysis, and interpretation of the study. All authors reviewed and approved the final manuscript.
